# Snake repellent safety and efficacy in Sub-Saharan Africa. What is the evidence?

**DOI:** 10.3389/fpubh.2026.1783520

**Published:** 2026-04-10

**Authors:** Lilian Nantume Wampande, Rosemary Nyariara Njoroge, Irene Wibabara, Anselme Shyaka, Ymkje Stienstra, Natnael Shimelash, Janna M. Schurer

**Affiliations:** 1 Center for One Health, University of Global Health Equity, Kigali, Rwanda; 2 Department of Internal Medicine/Infectious Diseases, University Medical Centre Groningen, University of Groningen, Groningen, Netherlands; 3 Centre for Snakebite Research and Interventions, Liverpool School of Tropical Medicine, Liverpool, United Kingdom; 4 School of Medicine, University of Global Health Equity, Kigali, Rwanda; 5 Department of Infectious Disease and Global Health, Cummings School of Veterinary Medicine at Tufts University, North Grafton, MA, United States

**Keywords:** snake repellents, efficacy, deterrence, naphthalene, essential oils, safety assessment, human and animal health, Africa

## Introduction

Sales of snake repellents are booming in Sub-Saharan Africa (SSA). A recently published study of commercial snake repellents available across six East-African countries (Burundi, Ethiopia, Kenya, Rwanda, Tanzania, Uganda) revealed 98 different products sold online and in agro-veterinary outlets (1). These ranged from chemical sprays, powders, and lotions to gum-traps and ultra-sonic devices. This is merely the tip of the iceberg. Far more so-called repellents and remedies are sold through non-conventional and unmonitored channels, such as local traditional healers (2).

Motivation to prevent snakebite envenoming (SBE) is high. Across SSA, SBE accounts for 12,290 (95%CI: 9,710–14,924) deaths and 14,766 (95%CI: 9,043–25,183) amputations per year, causing an annual burden of 1.03 million DALYs (95%CI: 0.80–1.28 million DALYs) (3). Those most affected are children and poor rural farmers who live furthest from health facilities but closest to snake habitats. Moreover, venomous snakes injure and kill livestock (4), impacting household income and food security. Clearly, prevention is crucial to achieving the World Health Organization (WHO) goal of halving SBE injuries and deaths by 2030 (5).

For those bitten, snake antivenoms (SAV) to treat envenomed people or livestock are often scarce, inappropriate, and/or expensive (6, 7). Across the continent, SSA receives only 2.5% of the SAV it needs, with frequent stock-outs across the private and public health sectors (8). In Rwanda, a single dose of SAV among uninsured individuals, costs on average 10 days of work, leaving some victims lingering with medical debts, stress and financial insecurity (6). Moreover, a recent WHO review of Good Manufacturing Practices among producers of African SAV identified widespread quality issues, clearing only one polyvalent SAV for use across SSA (9). In the absence of SAV, communities often defer to traditional healers for advice on how to repel snakes or treat SBE (2).

## Do snake repellents work?

**Figure 1:**
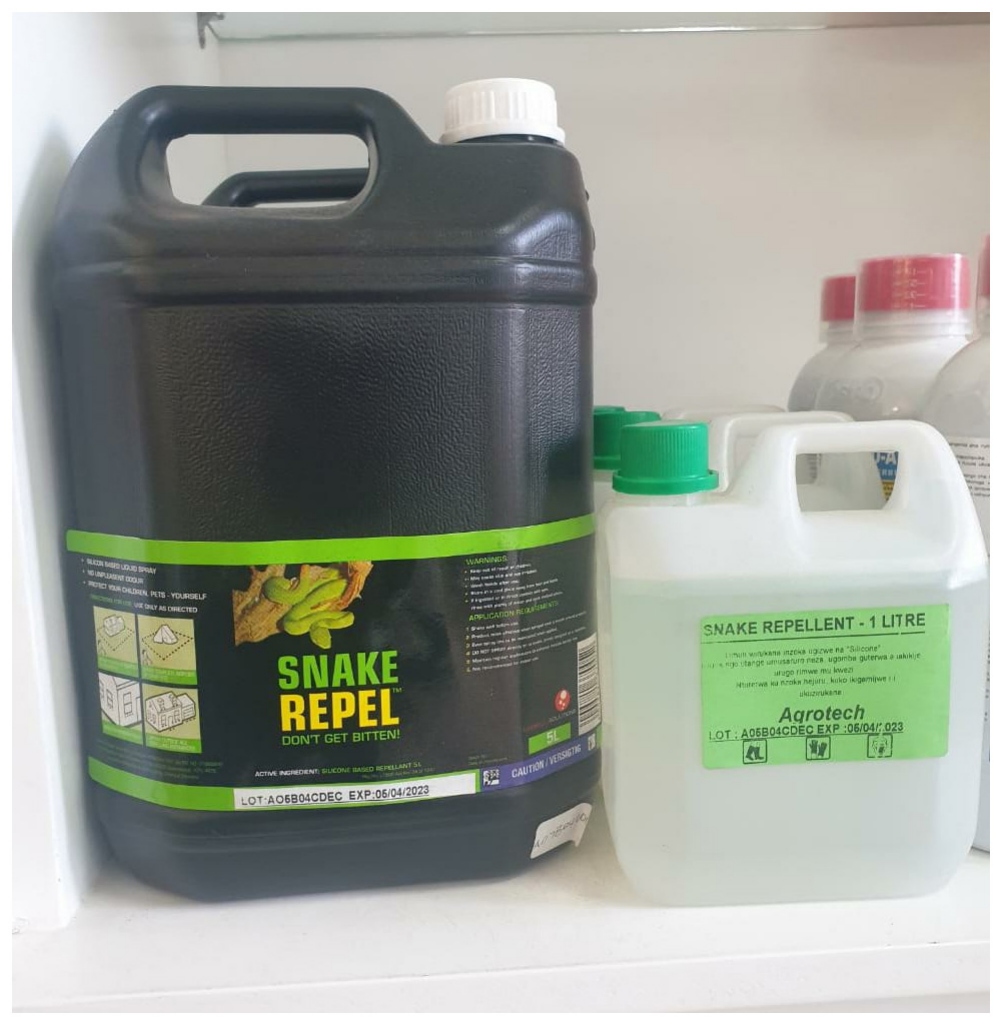
Chemical snake repellents sold in agro-chemical shop in Rwanda (Photo credit: Didier Usengimana, project field enumerator).

In the original study, a desk-based assessment of product ingredients was performed to evaluate safety and appropriateness of snake repellents being sold across 78 agro-veterinary outlets (1). Fifty-six chemical products, in the form of liquids, granules and powders (Figure 1), mostly containing essential oils (cinnamon, peppermint, cloves, and cedarwood) were found. Some products claimed efficacy against *Pantherophis obsoletus* (Black snake), *Pituophis catenifer sayi* (Bull snake), *Lampropeltis triangulum* (Milk snakes), and *Agkistrodon contortrix* (Copperhead); none of which are found on the African continent. Only one study in Africa has experimentally evaluated the efficacy of essential oils and other popular outdoor chemicals in driving away snakes in an outdoor real-world environment (10). None of the products demonstrated effectiveness. This contrasts with Brazil, where efficacy of olfactory repellents on *Bothrops moojeni* was demonstrated under controlled experimental conditions (11). The study demonstrated the potential of a natural non-toxic alternative for snake management. However, observation of snake behavior under a strict controlled environment may not fully replicate what happens in a real-world natural environment. Additionally, snakes' behavioral avoidance and irritation in the presence of natural product compounds may suggest a plausible chemosensory disruption mechanism of action, but does not necessarily translate into long-term removal of snakes.

The original study documented 42 mechanical products, including electric vibrators, ultra-sonic sound devices and glue traps (1). The solar powered ultra-sonic devices claimed to emit high-frequency waves that disrupted snake behavior by emitting sounds and vibrations through the soil. However, there is no scientific evidence to support this claim. The full methodology for evaluating repellent safety and efficacy is described in the original article (1).

## Are snake repellents safe?

More than one-third (42%) of the chemical repellents evaluated in the original study were not intended to be used as snake repellents (1). Such products included disinfectants like soap, tar acids and Kerol; fertilizers such as ammonium sulfate; and acaricides such as Mission 415EC (formulated to control diamondback moths in cabbages and/or ticks and mites in livestock). Misuse of acaricides has been linked to environmental degradation, increased prevalence of tick-borne diseases in humans and animals, and economic loss (12). Mothballs, which contain up to 99.95% of naphthalene or paradichlorobenzene, were also marketed as effective snake repellents (1). Naphthalene has a permissible exposure limit of 0.001% (13). Inhalation exposure above this level can be toxic to humans and animals, causing damage to the liver, kidney, skin, lungs, and nervous system (14). These ingredients can also release fumes that pollute air, leak into the soil, and contaminate ground water. From an environmental point of view, mechanical traps could be considered safer. However, there is no literature to support their efficacy in deterring or trapping snakes. Moreover, the traps are non-specific and pose a hazard to “unaware” humans such as children and non-target animals. Continued use of unproven products undermines integrated snake management approaches as these ineffective products create a false sense of security, leading to decreased vigilance, and increased risk of snakebites and the neglect of proven habitat modification methods. The WHO recommended long-term strategies on prevention focusing on education and environmental modification would benefit communities rather than relying on ineffective commercial repellents (15).

Snake repellent products often lack adequate labels (1, 16). Product labels are a legal requirement, and they convey important information about product name, manufacturer, ingredients list, storage conditions, usage instructions, expiration dates, and potential hazards, among others. In East Africa, it is currently not possible to fully evaluate the safety of snake repellents for people, environment, snakes, or other animals due to this gap in product labeling.

## Communication gaps and regulatory challenges

The sale of ineffective and possibly unsafe snake repellents in East Africa indicates serious gaps in effective One Health evidence-based regulation, coordination, and enforcement across public health, environment, and agricultural sectors. Moreover, publicly available resources on SBE prevention often omit advice related to the use of snake repellents in protecting farmers and their livestock. It is unclear how snake repellents are categorized to ensure appropriate risk management and consumer protection. In East Africa, countries can have numerous agencies responsible for the regulation and enforcement of agro-chemical policies, shaping how quality and safety standards are met. In Rwanda, these include the Rwanda Inspectorate, Competition and Consumer Protection Authority (RICA), which is responsible for registering and licensing all agrochemical traders; the Food and Agricultural Organization (FAO), which facilitates development of harmonized pesticide guidelines; and Rwanda Food and Drug Authority (FDA), which sets standards for quality, safety, and efficacy. It is not clear how such agencies coordinate their activities regionally.

The Mutual Recognition Agreement that sought to harmonize guidelines for veterinary medicines between East African Communities is a typical example of how joint enforcement can mitigate unwanted/sub-standard products in the region (17). A similar approach could benefit the local evaluation of snake repellents and antivenoms that currently lack standardized guidelines and monitoring systems.

## Way forward

There is lack of credible high-quality data in form of peer-reviewed, reproducible, and objective studies to support conclusions about the safety of snake repellents and their efficacy in protecting humans and livestock. With the current global focus on SBE, now is an opportune time to conduct research on these topics and to update public websites and learning materials to provide accurate information on reputable methods for snakebite prevention. Moreover, the dissemination of such information to diverse policy actors is needed to ensure that such stakeholders work collectively to remove inappropriate information and products from the public domain.
